# Carbonic Anhydrase Inhibitors rescue microvascular impairment and neuroinflammation in 3xTg AD mice

**DOI:** 10.1002/alz.093054

**Published:** 2025-01-03

**Authors:** Elisa Canepa, Nicole L Lemon, Micaelly Alves, Rafael Vazquez‐Torres, Maryam H. Abyaneh, Silvia Fossati

**Affiliations:** ^1^ Alzheimer’s Center at Lewis Katz School of Medicine, Temple University, Philadelphia, PA USA; ^2^ Lewis Katz School of Medicine, Temple University, Philadelphia, PA USA; ^3^ Temple University, Phialdelphia, PA USA; ^4^ Alzheimer’s Center Lewis Katz School of Medicine, Temple University, Philadelphia, PA USA

## Abstract

**Background:**

Alzheimer’s disease (AD) is characterized‐ at both early and late stages‐ by neurovascular impairment. In AD, dysfunctional cerebral microvasculature is accompanied by an inflammatory response, contributing to Aβ and tau accumulation, brain cell stress and death, impaired clearance of metabolic waste, BBB permeability, and ultimately leading to neuronal demise and cognitive impairment. We previously showed that Aβ peptides induce mitochondrial dysregulation and caspase‐mediated apoptosis in brain cells, including endothelial, glial, and smooth muscle cells. Moreover, we found FDA‐approved carbonic anhydrase inhibitors (CAIs), acetazolamide (ATZ) and methazolamide (MTZ), clinically used for non‐AD related conditions, effectively rescue these pathological events, and ameliorate cognitive impairment, brain amyloidosis, and vascular and glial fitness, in an AD/CAA (Cerebral Amyloid Angiopathy) *in vivo* model. We unveiled a mitochondrial isoform, Carbonic Anhydrase (CA‐VB), as a major player in Aβ‐mediated toxicity, with increased CA‐VB levels in AD/CAA mouse and human brains.

**Method:**

3xTg mice express three familial AD mutations: Swedish APP mutation (KM670/671NL), PSEN1 M146V mutation, and MAPT P301L mutation. Following 10 month‐CAI‐diet, mice were sacrificed at 16 months of age,. Brains were harvested and processed for biochemical and immunohistochemistry analysis.

**Result:**

CAIs ameliorate cognitive dysfunction**,** and significantly reduce hippocampal and cortical Aβ deposition and Thioflavin S positive deposits in 3xTg mice. Specifically in the hippocampus, CAIs rescue OXPHOS complexes dysregulated expression, reduce mitochondrial CA‐VB expression, and prevent caspase‐3 activation. In hippocampus and cortex, 3xTg present loss of PDGFRβ (microvascular marker) and increased gliosis (GFAP and IBA1), which are rescued by CAIs.

**Conclusion:**

CAIs show positive behavioral outcomes in 16‐month‐old 3xTg mice, and reduce brain amyloidosis, compared to untreated animals. In the hippocampus, CAIs foster microvascular health and reduce inflammatory state, and in the cortex, MTZ decreases microgliosis, unveiling cell specific CAI effects underlying Aβ deposition reduction observed in both areas. This work points to CAIs as a potential therapeutic strategy to treat brain microvascular impairment and neuroinflammation, preventing mitochondria‐mediated cell stress and death.

